# Non-syndromic cardiac progeria in a patient with the rare pathogenic p.Asp300Asn variant in the *LMNA* gene

**DOI:** 10.1186/s12881-017-0480-x

**Published:** 2017-10-18

**Authors:** Ali J. Marian

**Affiliations:** 1 Center for Cardiovascular Genetics, Institute of Molecular Medicine, University of Texas Health Sciences Center at Houston, 6770 Bertner Street, DAC900, Houston, TX 77030 USA; 2 Texas Heart Institute, 6770 Bertner Street, DAC900, Houston, TX 77030 USA

**Keywords:** Progeria, Lamin A/C, Degenerative heart disease, Cardiomyopathy, Valvular disease, Genetics, Case report

## Abstract

**Background:**

Mutations in *LMNA* gene, encoding Lamin A/C, cause a diverse array of phenotypes, collectively referred to as laminopathies. The most common manifestation is dilated cardiomyopathy (DCM), occurring in conjunction with variable skeletal muscle involvement but without involvement of the coronary arteries. Much less commonly, *LMNA* mutations cause progeroid syndromes, whereby an early-onset coronary artery disease (CAD) is the hallmark of the disease. We report a hitherto unreported compound cardiac phenotype, dubbed as “non-syndromic cardiac progeria”, in a young patient who carried a rare pathogenic variant in the *LMNA* gene and developed progressive degeneration of various cardiac structures, as seen in the elderly. The phenotype resembled the progeroid syndromes, except that it was restricted to the heart and did not involve other organs.

**Case presentation:**

The patient was a well-developed Caucasian female who presented at age 29 years with an acute myocardial infarction (MI) and was found to have extensive CAD. She had none of the conventional risk factors for atherosclerosis. She underwent coronary artery bypass surgery but continued to require multiple percutaneous coronary interventions for symptomatic obstructive coronary lesions. During the course of next 10 years, she developed mitral regurgitation, degenerative mitral and aortic valve diseases, atrial flutter, and progressive conduction defects. She died from progressive heart failure with predominant involvement of the right ventricle and severe tricuspid regurgitation. Cardiac phenotype in this young patient resembled degenerative cardiac diseases of the elderly and the progeroid syndromes. However, in contrast to the progeroid syndromes, the phenotype was restricted to the heart and did not involve other organs. Thus, the phenotype was dubbed as a non-syndromic cardiac progeria.

Genetic screening of several cardiomyopathy genes, including LMNA, which is a causal gene for progeroid syndromes, led to identification of a very rare pathogenic p.Asp300Asn variant in the *LMNA* gene.

**Conclusions:**

We infer that the *LMNA* p.Asp300Asn mutation is pathogenic in non-syndromic cardiac progeria. Mutations involving codon 300 in the *LMNA* gene have been associated with progeroid syndromes involving multiple organs. Collectively, the data provide credence to the causal role of p.Asp300Asn mutation in the pathogenesis of non-syndromic cardiac progeria.

**Electronic supplementary material:**

The online version of this article (10.1186/s12881-017-0480-x) contains supplementary material, which is available to authorized users.

## Backgrounds

Aging is associated with a progressive functional decline of multiple organ, including the heart. In the heart, aging commonly manifests with cardiac dysfunction, an impaired chronotropic response, atrial fibrillation, conduction defects, degenerative valvular disease, and coronary atherosclerosis, as well as vascular calcification. The prevalence of these phenotypes, which are typically absent in the young individuals, increases markedly in the elderly. For example, heart failure, with the exception of familial cardiomyopathies, predominantly affects the elderly [1]. Likewise, cardiac conduction defects and arrhythmias are predominantly diseases of the elderly [2]. Similarly, degenerative mitral and aortic valve diseases are also almost exclusive diseases of the elderly, and seldom seen in young individuals except in those patients with a bicuspid aortic valve [3].

The *LMNA* gene encodes lamin A (and its isoforms C, C2, and Δ10), which is an inner nuclear membrane protein ubiquitously expressed in almost all differentiated cells, including the cardiac cells [4, 5]. Mutations in the *LMNA* gene cause a diverse array of phenotypes, which are collectively referred to as laminopathies [6]. In the heart, *LMNA* mutations cause two distinct sets of phenotypes, involving primarily either the myocardium or the coronary arteries, the latter in the context of progeroid syndromes. Most commonly *LMNA* mutations cause dilated cardiomyopathy (DCM), with variable skeletal muscle involvement [7, 8]. The phenotype is typically associated with early conduction defects and refractory heart failure [7–11]. Such patients typically do not exhibit premature coronary artery disease (CAD) and myocardial infarction (MI). Less commonly, however, patients with *LMNA* mutations present with an early onset CAD and premature MI, typically in the context of progeroid syndromes, such as Hutchinson-Gilford Progeria Syndrome (HGPS) and atypical Werner syndrome, which typically involve multiple organs [12, 13].

Phenotypic manifestations of progeroid syndromes is diverse and include impaired growth, alopecia, skin sclerosis, bone abnormalities, subcutaneous fat redistribution, and cardiovascular complications, including atherosclerosis and myocardial infarction, reflective of involvement of multiple cell types and organs [14, 15]. Cardiovascular complications, including advanced atherosclerosis and MI, are the main causes of death in patients with HGPS [16]. We report a young patient who carried a rare missense mutation in the *LMNA* gene and presented with the phenotype that resembled the progeroid syndromes. However, in contrast to the progeroid syndromes, the phenotype was restricted to the heart and did not involve other organs. The predominant phenotype was premature CAD and MI, but also included degenerative valvular disease, conduction defect, and premature death due to refractory right heart failure.

## Case presentation

The patient was a fully developed (height: 165 cm, weight: 110 lbs) Caucasian female who first presented with an acute MI at age 29 years. She did not have a family history of premature CAD and did not have any of the conventional risk factors for MI (dyslipidemia, smoking, diabetes mellitus, and systemic arterial hypertension). She underwent cardiac catheterization and coronary angiography and was found to have advanced CAD. The early onset of CAD in this patient is in contrast to the typical presentation of CAD in the 6th and 7th decades of life in the general population. The presentation is also in discord with that of CAD in patients with HGPS, who typically suffer from CAD and MI in childhood [16]. The patient underwent coronary artery bypass surgery with implantation of the left internal mammary artery to left anterior descending coronary artery and a vein graft to left circumflex coronary artery. The time course of the medical problems and interventions are listed in Table 1 and the list of medication in Additional file 1: Table S1. In brief, during the course of next 10 years, she developed mitral valve regurgitation; requiring surgical repair, chest pain due to obstructive coronary lesions; requiring multiple percutaneous coronary interventions, degenerative mitral and aortic valve diseases; requiring replacement of both valves, and atrial flutter/fibrillation along with conduction defect; requiring catheter ablation and a permanent pacemaker implantation. She developed progressive heart failure, predominantly involving the right ventricle with severe tricuspid regurgitation, and died a year later at the age of 40 years. During the last hospital admission, she was evaluated for heart transplantation. Notable cardiovascular test/procedures findings are summarized in Table 2.

**Table 1:** Time course of the phenotype in the proband

Year	Age	Phenotype	Intervention
1994	29	Acute myocardial infarction	Coronary Artery bypass surgery
1995	30	Mitral regurgitation	Mitral valve repair surgery
2001	36	Acute myocardial infarction	Percutaneous coronary interventions
2001	36	Sick Sinus Syndrome	Permanent pacemaker implantation
2001	36	Aortic and mitral valves stenosis/regurgitation	Aortic valve replacement Mitral valve replacement
2003	38	Atrial flutter	Catheter ablation
2004	39	Acute myocardial infarction	Percutaneous coronary interventions, placement of 2 stents
2004	39	Refractory right heart failure with severe tricuspid regurgitation	Transplant evaluation
2005	40	Death

**Table 2:** Diagnostic tests results during last hospital admission

Test/Procedure	Findings
12-lead electrocardiogram	• Dual chamber AV-sequential paced rhythm • Left bundle branch QRS morphology • Isolated premature ventricular contractions
Transthoracic echocardiogram	• Normal left ventricular size • Mildly depressed left ventricular function • Moderately to severely enlarged RV • Normally functioning prosthetic aortic and mitral valves • Estimated pulmonary artery systolic pressure > 40 mmHg
Trans-Esophageal echocardiogram	• Dilated tricuspid annulus (4.2 cm) • Non-coapting tricuspid valve leaflets • Severe tricuspid regurgitation • Enlarged right atrium
Adenosine myocardial perfusion tomography	• Left ventricular ejection fraction: 49% • Moderately hypokinetic left ventricle • Perfusion defect: 17% • Fixed perfusion defect: 6% • Reversible perfusion defect 11% in the left anterior descending coronary territory
Right heart catheterization	• Right atrial pressure: 17 mmHg • Right ventricular pressure: 44/3 mmHg (mean 22) • Pulmonary capillary wedge pressure: 20 mmHg • Cardiac output: 4.6 L/min (Cardiac index: 3.0 L/min/m^2^)
Carotid Doppler	• Less than 50% stenosis in both carotids
Endomyocardial biopsy (right ventricle)	• Hypertrophic cardiac fibers • Enlarged myocyte nuclei • Negative for amyloid (Congo stain) • No evidence of myocarditis

During evaluation for cardiac transplantation other organs were also evaluated. She had no features to suggest a systemic progeroid syndrome and had only a mild pre-renal azotemia and a restrictive physiology on a pulmonary function test. These abnormalities were considered to be secondary to heart failure and not features of progeroid syndromes. Computerized tomography of chest, abdomen, and pelvis were unremarkable, except for abdominal aortic atherosclerosis and evidence of right heart failure (enlarged inferior vena cava and hepatic vein along with a congested liver, and ascites). Her lipid profile was notable for a total cholesterol of 130 mg/dL (desirable level: <200 mg/dL), HDL-C 24 mg/dL (normal range: 40 to 60 mg/dL), LDL-C of 75 mg/dL (optimal level < 100 mg/dL), and triglycerides of 155 mg/dL (normal levels <150 mg/dL). Her other laboratory blood tests were remarkable for an elevated blood homocysteine concentration of 27.2 umol/L (normal range: 4.0 to 10.0 umol/L), anemia of chronic disease (Hg: 9.7 g/dL, normal range: 12.0 -16.0 g/dL), and an elevated B-type natriuretic peptide level of 467 pg/mL (normal range: 0-100 pg/mL), consistent with heart failure. None of the above laboratory values were specific to progeroid syndromes. Considering the constellation of multiple cardiovascular phenotypes, typically observed in the elderly and in the progeroid syndromes, and given the absence of a progeroid phenotype in other organs, the term non-syndromic cardiac progeria was coined to describe the phenotype in the index case.

**Fig. 1:**
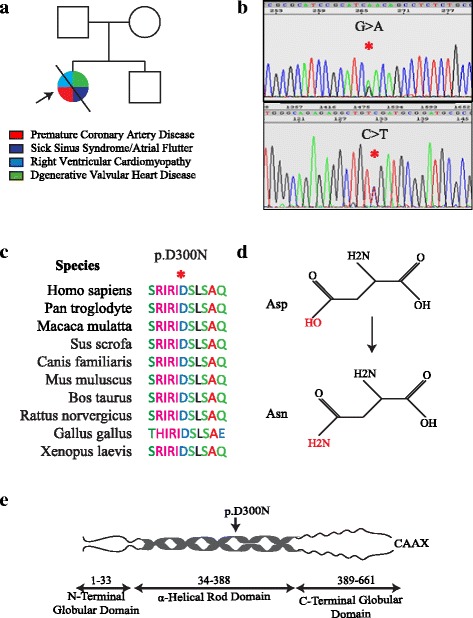
Identification of p.Asp300Asn Mutation in the *LMNA* Gene. **a** Pedigree of the proband containing the phenotypic data. Square box and circle represent male and female members respectively. Full circle indicates an affected member. The / symbol indicates a deceased individual. **b** Electrophoregrams showing the presence of the mutation in the sense and anti-sense directions. **c** Evolutionary conservation of the Asp300 amino acid across several species. **d** The change in the structure of the involved amino acid from a hydroxyl group to an amine group. **e** Location of the p.Asp300Asn mutation in the LMNA protein, which is the site of LMNA dimerization

In view of the well-established role of the *LMNA* gene in progeroid syndromes, the *LMNA* gene along with several genes commonly associated with cardiomyopathies, namely, *MYH7* (myosin heavy chain 7), *MYPBC3* (myosin binding protein C3), *TNNT2* (cardiac troponin T), *TNNI3* (cardiac troponin I), *ACTC1* (cardiac α-actin), and *TPM1* (α-tropomyosin) were sequenced using the Big Dye Terminator Cycle Sequencing Ready Reaction Kit on an ABI Genetic Analyzer 3730xl (Applied Biosystems, Foster City, CA), as published [17–19]. Both sense and anti-sense DNA strands of all exons and the exon-intron boundaries were sequenced. The sequence output was analyzed using Variant Reporter software (Applied Biosystems) and compared the sequence with the corresponding reference GenBank sequence of each gene. A rare *LMNA* gene p.Asp300Asn missense variant was identified in the proband (Fig. 1). No pathogenic variant in other genes was detected. The proband’s parents and brother either could not be reached or decided not to participate in the genetic studies. The p.Asp300Asn variant was absent in the gnomAD database (http://gnomad.broadinstitute.org/gene/ENSG00000160789.). It is predicted to be pathogenic by multiple computational algorithms (Polyphen2 score: 0.995, SIFT score: 0.03, Mutation taster score: 1, CADD_Phred score: 27.6). The mutation affected the coiled coil structure in the rod domain of the LMNA protein, which is involved in binding to lamin A/C dimers and partners.

## Discussion and conclusions

The p.Asp300Asn was identified a few months after patient’s death about 10 years ago. However, it was not reported because of the uncertainty in unambiguous ascertainment of its causality in a single case, despite its pathogenic nature and biological plausibility. A recent report of detection of the p.Asp300Asn mutation in a Japanese patient who exhibited atypical progeroid/Werner syndrome involving multiple organs [20], provided strong support to the causal role of this mutation in the patient with non-syndromic cardiac progeria. In principle, detection of a rare pathogenic variant in two independent individuals with a similar phenotype provides strong evidence of pathogenicity of the variant in the phenotype of interest [21]. Moreover, a different missense mutation involving the amino acid 300 (p.Asp300Gly) in the LMNA protein has been associated with an autosomal dominant late-onset cardiocutaneous progeria [22]. The skin phenotype associated with the p.Asp300Gly included early hair loss and premature graying, whereas the cardiac involvement was comprised of accelerated atherosclerosis, calcific valve disease, DCM, and MI, the latter leading to premature death at age 44 years [22]. The phenotype in our patient who carried the p.Asp300Asn is unique and distinct from other progeroid syndromes, as it does not involve other organs. It solely restricted to the heart where it affects multiple structures, including coronary arteries, aortic and mitral valves, conduction system, and the right ventricle. Based on the data presented in this report and the existing data on progeroid syndromes caused by the *LMNA* mutations involving codon 300, we infer that the p.Asp300Asn is responsible for non-syndromic cardiac progeria in the index patient presented in this report.

Thus, the patient exhibits the novel phenotype of non-syndromic cardiac progeria, characterized by degenerative disease of multiple cardiac structures, including the coronary arteries, valves, the conduction system, and a lesser extent the myocardium, likely caused by a rare pathogenic variant p.Asp300Asn in the *LMNA* gene.

## Additional file

Additional file 1: Table S1. Patient’s medication list on last admission. The table lists patient’s medications. (DOCX 57 kb)

## Abbreviations

ACTC1: Cardiac α-actin
CAD: Coronary artery disease
DCM: Dilated Cardiomyopathy
HDL-C: High-density lipoprotein –cholesterol
HGPS: Hutchinson-gilford progeria syndrome
LDL-C: Low-density lipoprotein-cholesterol
LMNA: Lamin A/C
MI: Myocardial infarction
MYH7: Myosin heavy chain 7
MYPBC3: Myosin binding protein C3
TNNI3: Cardiac troponin I
TNNT2: Cardiac troponin T
TPM1: α-tropomyosin
