# Supplementary material for: Non-syndromic cardiac progeria in a patient with the rare pathogenic p.Asp300Asn variant in the LMNA gene
Source: BMC Med Genet. 2017 Oct 18;18:116. doi: 10.1186/s12881-017-0480-x (PMC5648416; doi:10.1186/s12881-017-0480-x)
Supplement: Additional file 1: Table S1. — Patient’s medication list on last admission. The table lists patient’s medications. (DOCX 57 kb) [file 12881_2017_480_MOESM1_ESM.docx]

**Additional file: Table S1. Patient’s medication list on last admission**

| **Medication** | **Dose** | **Route** | **Frequency** |
| --- | --- | --- | --- |
| Aspirin | 81 mg | Oral | Daily |
| Coumadin | 3 mg | Oral | Daily |
| Diltiazem | 300 mg | Oral | Daily |
| Protonix | 40 mg | Oral | Twice a day |
| Zetia | 10 mg | Oral | Daily |
| Isosorbide mononitrate | 120 mg | Oral | Daily |
| Demadex | 40 mg | Oral | Daily |
| Carvidelol | 3.125 mg | Oral | Twice a day |
| Furosemide | 40 mg | Oral | Daily |
| Norvasc | 5 mg | Oral | Daily |
| Foltex | 1 mg | Oral | Daily |
| Ambein | 10 mg | Oral | Per night as needed |
| As Acetaminophen | 650 mg | Oral | every 6 hours as needed |
